# Soluble Epoxide Hydrolase Inhibition for Ocular Diseases: Vision for the Future

**DOI:** 10.3389/fphar.2019.00095

**Published:** 2019-02-07

**Authors:** Bomina Park, Timothy W. Corson

**Affiliations:** ^1^Department of Ophthalmology, Eugene and Marilyn Glick Eye Institute, Indiana University School of Medicine, Indianapolis, IN, United States; ^2^Department of Pharmacology and Toxicology, Indiana University School of Medicine, Indianapolis, IN, United States; ^3^Department of Biochemistry and Molecular Biology, Indiana University School of Medicine, Indianapolis, IN, United States

**Keywords:** soluble epoxide hydrolase, small molecule inhibitor, age-related macular degeneration, diabetic retinopathy, diabetic keratopathy, uveitis, angiogenesis

## Abstract

Ocular diseases cause visual impairment and blindness, imposing a devastating impact on quality of life and a substantial societal economic burden. Many such diseases lack universally effective pharmacotherapies. Therefore, understanding the mediators involved in their pathophysiology is necessary for the development of therapeutic strategies. To this end, the hydrolase activity of soluble epoxide hydrolase (sEH) has been explored in the context of several eye diseases, due to its implications in vascular diseases through metabolism of bioactive epoxygenated fatty acids. In this mini-review, we discuss the mounting evidence associating sEH with ocular diseases and its therapeutic value as a target. Substantial data link sEH with the retinal and choroidal neovascularization underlying diseases such as wet age-related macular degeneration, retinopathy of prematurity, and proliferative diabetic retinopathy, although some conflicting results pose challenges for the synthesis of a common mechanism. sEH also shows therapeutic relevance in non-proliferative diabetic retinopathy and diabetic keratopathy, and sEH inhibition has been tested in a uveitis model. Various approaches have been implemented to assess sEH function in the eye, including expression analyses, genetic manipulation, pharmacological targeting of sEH, and modulation of certain lipid metabolites that are upstream and downstream of sEH. On balance, sEH inhibition shows considerable promise for treating multiple eye diseases. The possibility of local delivery of inhibitors makes the eye an appealing target for future sEH drug development initiatives.

## Introduction

**FIGURE 1:**
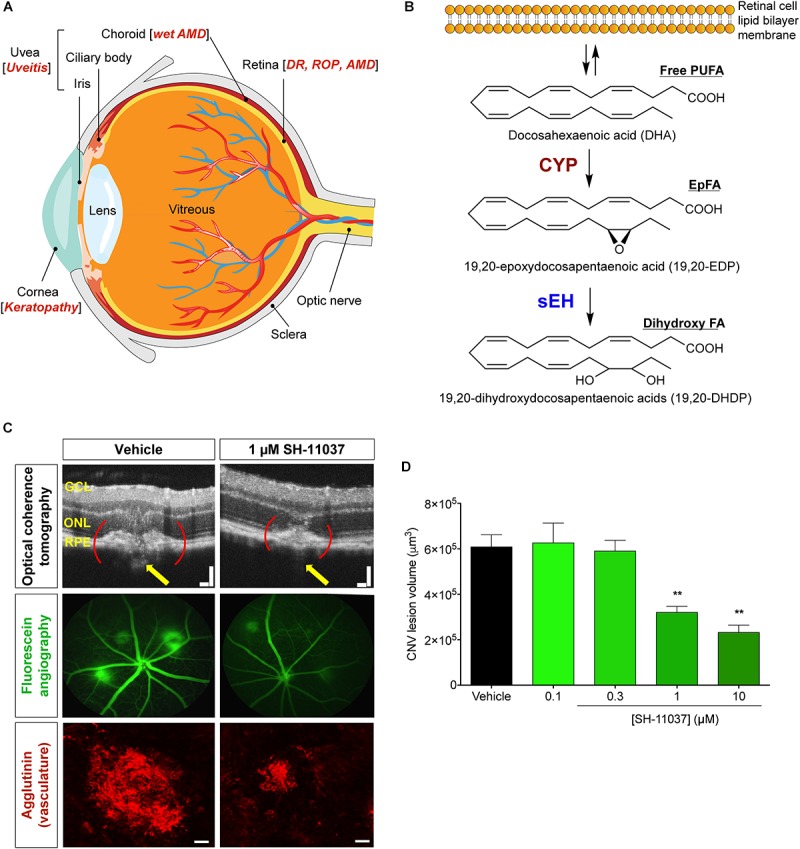
Schematic representation of the eye and PUFA metabolism by the CYP-sEH pathway, and effects of sEH inhibition *in vivo*. **(A)** Common eye diseases and associated structures in the human eye. **(B)** Retina has the highest concentration of ω-3 docosahexaenoic acid of all fatty acids. The regulation of bioactive epoxygenated fatty acids takes place through production by cytochrome P450 epoxygenase and degradation by soluble epoxide hydrolase (sEH). ω-3 fatty acids shown; the same pathway acts on ω-6 fatty acids. **(C,D)** sEH inhibitor SH-11037 dose-dependently suppresses L-CNV lesion volumes. Figure modified from Sulaiman et al. (2016). **(C)** Representative imaging data. Optical coherence tomography obtained 7 days post-laser. Yellow arrows highlight regions containing CNV. Scale bars = 100 μm. Fluorescein angiography images 14 days post-laser and confocal microscopy images for agglutinin-stained CNV lesions 14 days post-laser. Scale bars = 50 μm. **(D)** Quantification of CNV lesion volumes from Z-stack images at day 14 using ImageJ software. ^∗∗^*P* < 0.01, one-way ANOVA, Tukey’s *post hoc* tests, Mean ± SEM, *n* = 12 eyes/treatment. AMD, age-related macular degeneration; CYP, cytochrome P450 epoxygenase; DR, diabetic retinopathy; EpFA, epoxygenated fatty acid; FA, fatty acid; PUFA, polyunsaturated fatty acid; ROP, retinopathy of prematurity; sEH, soluble epoxide hydrolase.

Visual impairment and blindness from ocular diseases can profoundly compromise patients’ quality of life, and imposes a substantial economic burden of $35.4 billion per year in the United States (Rein et al., 2006). The development of anti-vascular endothelial growth factor (anti-VEGF) therapies has advanced treatment for neovascular eye diseases, but these drugs have drawbacks and there is a lack of pharmacotherapies for other ocular diseases (Figure 1A) despite consistent and intense expansion in market potential for ocular therapeutics (Kompella et al., 2010). Understanding the mediators involved in pathophysiology and identification of therapeutic targets and inhibitors are necessary in order to address these unmet therapeutic needs.

There is a growing awareness of the importance of bioactive lipid metabolism to ocular structure, function, and pathology. Especially, the unique lipid profile of the retina gives an outsized role for docosahexaenoic acid (DHA, 22:6 ω-3) and DHA-derived polyunsaturated fatty acid (PUFA) metabolites in the eye (Figure 1B). DHA is a major structural component of the membrane phospholipids in the retina (Querques et al., 2011), constituting 50–60% of the total fatty acids in the outer segments of photoreceptors (Stinson et al., 1991; Bush et al., 1994; Stillwell and Wassall, 2003), in contrast to most tissues that contain only a small portion (∼5%) of their fatty acids as DHA. In parallel with the arachidonic acid (ARA, 20:4 ω-6) cascade, the metabolism of DHA involves three branches of oxylipin synthesis enzymes: cyclooxygenase (COX), lipoxygenase (LOX), and cytochrome P450 (CYP) epoxygenases, of which the CYPs are responsible for generating bioactive epoxygenated fatty acids (EpFAs) (Morisseau et al., 2010; Zhang et al., 2013; Malamas et al., 2017). EpFAs like epoxyeicosatrienoic acids (EETs) from ω-6 ARA and epoxydocosapentaenoic acids (EDPs) from ω-3 DHA have garnered much attention in vascular disorders due to their vasodilatory and anti-inflammatory properties (Ye et al., 2002; Zhang et al., 2014; Capozzi et al., 2016).

Epoxygenated fatty acids are physiologically unstable because they are rapidly metabolized, mainly by soluble epoxide hydrolase (sEH) (Chacos et al., 1983; Figure 1B). sEH, encoded by the *EPHX2* gene, has a C-terminal hydrolase function that acts on lipid epoxides, plus a poorly studied N-terminal phosphatase activity (Harris and Hammock, 2013). Inhibition of sEH stabilizes EpFAs, enhancing their biological activities, which vary among EpFAs derived from ω-6 and ω-3 PUFAs. EETs and EDPs have vasodilatory (Oltman et al., 1998; Zhang et al., 2001; Ye et al., 2002) and analgesic effects, reducing inflammatory pain (Inceoglu et al., 2008; Morisseau et al., 2010; Wagner et al., 2014). But they have contradictory effects on angiogenesis: EETs usually have proangiogenic effects depending on the experimental context (Pozzi et al., 2005; Michaelis et al., 2008; Xu et al., 2013), whereas EDPs have antiangiogenic effects (Zhang et al., 2013; Capozzi et al., 2014; Hasegawa et al., 2017; Hu et al., 2017). Moreover, sEH mediated metabolism of EpFAs produces lipid diols like dihydroxydocosapentaenoic acids (DHDP) (Figure 1B). Thus, sEH inhibition can result in tissue specific effects by modulating different classes of EpFAs depending on the abundance of individual PUFAs in the given tissue.

Genetic manipulation of CYP/sEH expression and small molecule mediated targeting of sEH have allowed investigation of the role of EpFAs in eye diseases, in particular diseases mediated by inflammation and angiogenesis. Through the metabolism of bioactive EpFAs and production of corresponding diols, sEH plays a role in the regulation of angiogenesis and inflammation relevant to the pathogenesis of numerous eye diseases. This mini-review discusses sEH as a therapeutic target for eye diseases and the role of PUFA metabolites of CYP and sEH in ocular neovascularization and other ocular disorders.

## sEh and Neovascular Eye Diseases

Ocular neovascularization (abnormal angiogenesis) is a prominent feature of blinding eye diseases including proliferative diabetic retinopathy (PDR), retinopathy of prematurity (ROP), and neovascular “wet” age-related macular degeneration (wet AMD) (Das and McGuire, 2003; Figure 1A). The new blood vessels that form in these diseases are leaky and prone to rupture, thereby causing vascular leakage, scarring, and even retinal detachment that can lead to permanent vision loss (Hageman et al., 2008). Efforts to treat neovascular eye diseases are hampered by resistance or refractory response to the current standard of care, anti-VEGF therapies (Lux et al., 2007). Therefore, identification of novel therapeutic targets and inhibitors is needed to address the unmet needs in antiangiogenic treatment. Metabolites of ω-3 PUFAs from the CYP-sEH pathway are emerging as important mediators of angiogenesis (Shao et al., 2014; Yanai et al., 2014; Gong et al., 2016a,b).

### EpFAs and Angiogenesis

Epoxydocosapentaenoic acids are anti-angiogenic *in vitro*: all the chemically stable EDP regioisomers inhibited VEGF-induced angiogenesis in a Matrigel plug assay (Zhang et al., 2013). Of note, 19,20-EDP, which is the least efficient substrate for sEH, therefore most abundant isomer (Zhang et al., 2014), had no effect on endothelial cell proliferation but strongly inhibited human umbilical vein endothelial cell tubular network formation and migration and weakly inhibited matrix metalloproteinase 2 (MMP-2) activity via a VEGF receptor 2 dependent manner (Zhang et al., 2013), although the exact mechanism through which EDPs crosstalk with VEGF signaling remains to be clarified.

ω-3 EpFAs also have anti-inflammatory and anti-angiogenic effects in animal models of ocular angiogenesis. Dietary intake of ω-3 PUFAs but not ω-6 PUFAs reduces murine laser-induced choroidal neovascularization (L-CNV) (Yanai et al., 2014), a widely used model in which laser ruptures Bruch’s membrane, resulting in angiogenesis from the choroid into the subretinal space. This recapitulates key features of wet AMD and serves as a model in which to test anti-angiogenic therapies (Lambert et al., 2013).

Dietary intake of ω-3 PUFAs in mice substantially enhanced levels of 17,18-epoxyeicosatetraenoic acid (EEQ) and 19,20-EDP in the serum lipid profile. However, it did not increase levels of EDPs in the retinal lipid profile. Interestingly, dietary intake of 17,18-EEQ or 19,20-EDP also suppressed CNV, suggesting that the protective effect of ω-3 PUFAs against CNV could be mediated by its downstream epoxy metabolites that are generated by CYP. In addition, dietary ω-3 PUFAs interfered with leukocyte invasion into the CNV lesions, while ω-6 PUFA did not (Yanai et al., 2014). These effects were associated with anti-inflammatory properties of EpFAs. Specifically, they modulated leukocyte rolling velocity by changing the expression of adhesion molecules on the surfaces of leukocytes and in the CNV lesions (Hasegawa et al., 2017). In transgenic mice overexpressing CYP2C8 in endothelial cells, the dietary intake of ω-3 PUFAs, which increased production of 17,18-EEQ and 19,20-EDP in serum, reduced CNV lesions (Hasegawa et al., 2017). Likewise, dietary ω-3 PUFAs reduced CNV lesions in *Ephx2*^-/-^ mice. These mice also had increased plasma levels of 17,18-EEQ and 19,20-EDP, since sEH-dependent degradation of these epoxides into corresponding diols was blocked. In contrast, dietary ω-3 PUFAs did not confer inhibitory effects on CNV in mice overexpressing sEH.

The relevance of sEH to CNV was further supported by our recent study that showed an increase in the expression of sEH in the eyes of L-CNV mice and human wet AMD patients (Sulaiman et al., 2018). Interestingly, sEH was upregulated in the photoreceptors, and ocular enzymatic activity of sEH was increased upon L-CNV induction in adult mice. The lipid profile of retina/choroidal tissue of the mice revealed that the ratio of 19,20-EDP to 19,20-DHDP was significantly reduced in L-CNV, suggesting enhanced sEH activity (Sulaiman et al., 2018). In the developing mouse retina, sEH is highly expressed in Müller glia, and DHDP produced by sEH contributes to retinal angiogenesis (Hu et al., 2014). Müller glia span the entire retina radially, providing structural and metabolic support for retinal neurons (Reichenbach and Bringmann, 2013). The Müller cell specific knockout of sEH or systemic deletion of sEH significantly impaired developmental retinal angiogenesis and altered the retinal lipid profile. The level of 19,20-DHDP was significantly reduced in the retina of *Ephx2*^-/-^ mice. Intravitreal injection of 19,20-DHDP rescued impaired retinal angiogenesis in *Ephx2*^-/-^ mice. 19,20-DHDP was also found to be a signaling molecule, downregulating the endothelial Notch signaling pathway by inhibiting presenilin-1 dependent γ-secretase activity, which is required for release of the Notch intracellular domain (Hu et al., 2014). Interestingly, the crosstalk between Notch and VEGF pathways in angiogenesis has been reported in numerous studies, where activation of Notch signaling modulates VEGF signaling (Hellström et al., 2007; Li and Harris, 2009). Given this, inhibition of sEH not only stabilizes the anti-angiogenic and anti-inflammatory ω-3 EpFAs, but also inhibits production of pro-angiogenic DHDP.

### Small Molecule sEH Inhibition and Ocular Angiogenesis

Targeting sEH with small molecule inhibitors effectively reduces ocular angiogenesis (Table 1). SH-11037, a synthetic homoisoflavonoid that we developed in cell-based assays and subsequently identified as an sEH inhibitor (Sulaiman et al., 2018), effectively blocked key angiogenic properties of human retinal endothelial cells (HRECs) – proliferation, migration and tube formation – without inducing cell death (Basavarajappa et al., 2015). As well, SH-11037 reduced angiogenesis in an *ex vivo* choroidal sprouting assay and inhibited developmental ocular angiogenesis in zebrafish larvae (Sulaiman et al., 2016). Local application of SH-11037 (1 μM) into the eye via intravitreal injection significantly suppressed CNV lesions (Sulaiman et al., 2016; Figure 1C,D) and was also effective in reducing retinal neovascularization in the oxygen-induced retinopathy (OIR) model (Basavarajappa et al., 2015), in which neonatal mouse pups are subjected to hyperoxia during their developmental retinal vascularization, causing ischemia-induced angiogenesis on return to normoxia (Scott and Fruttiger, 2009; Kim et al., 2016). Structural, morphological and vascular examination of retina and electroretinography showed that up to 100 μM intravitreal SH-11037 does not exert ocular toxicity (Sulaiman et al., 2016). Excitingly, SH-11037 also synergized with anti-VEGF therapy to reduce L-CNV (Sulaiman et al., 2016).

**Table 1:** Soluble epoxide hydrolase inhibitors tested in ocular disease animal models.

sEH inhibitor (Reference)	Routes	Dose	Model	Reference
***t*-AUCB (UC1471)** (Hwang et al., 2007) 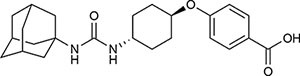	Oral	2 mg/L	Mouse diabetic retinopathy, sEHI in drinking water for 10 months	Hu et al., 2017


	Intravitreal	1–10 μM	Mouse L-CNV, single sEHI injection	Sulaiman et al., 2018
	Intraperitoneal	2 mg/kg	Mouse neonatal retinal angiogenesis, twice a day sEHI injections for postnatal (P) days 1–4	Hu et al., 2014
	Subconjunctival	10 nM	Mouse diabetic keratopathy, single sEHI delivery	Sun et al., 2018

**TPPU (UC1770)** (Rose et al., 2010) 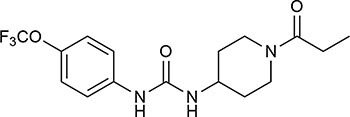	Intraperitoneal	0.3 mg/kg	Mouse OIR, daily sEHI injections from P12-P16	Gong et al., 2016a


			Mouse L-CNV, daily sEHI injections from day 0–6	
	Oral	1 mg/kg	Mouse L-CNV, daily sEHI delivery from 3 days before CNV induction to day 7	Hasegawa et al., 2017

**SH-11037** (Basavarajappa et al., 2015) 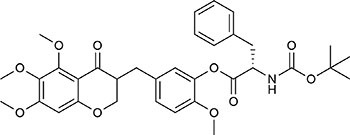	Intravitreal	0.1–100 μM	Mouse ocular toxicity	Sulaiman et al., 2016


		1–10 μM	Mouse L-CNV, single sEHI injection	
	Systemic (in larvae water)	1–10 μM	Ocular angiogenesis in zebrafish larvae, sEHI treatment 2–5 days post fertilization (dpf)	
	Intravitreal	1 μM	Mouse OIR, single sEHI injection	Basavarajappa et al., 2015

**“Compound 7”** (Shen et al., 2009) 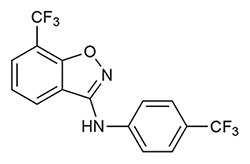	Intravitreal	10–30 μM	Mouse L-CNV, single sEHI injection	Sulaiman et al., 2018

***t*-TUCB (UC1728)** (Hwang et al., 2007) 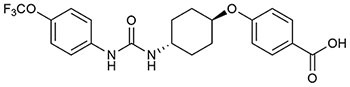	Subcutaneous	3 mg/kg	Rabbit LPS-induced uveitis, daily sEHI injections	McLellan et al., 2016

**GSK2256294A** (Podolin et al., 2013) 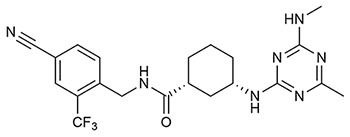	Subconjunctival	10 nM	Mouse diabetic keratopathy, single sEHI delivery	Sun et al., 2018


*L-CNV, laser-induced choroidal neovascularization; LPS, lipopolysaccharide; OIR, oxygen-induced retinopathy; P, postnatal; sEHI, soluble epoxide hydrolase inhibitor.*

Intravitreal injection of other known sEH inhibitors *t*-AUCB (1–10 μM) and “compound 7” (10–30 μM) also suppressed L-CNV lesions (Sulaiman et al., 2018; Table 1). Moreover, intravitreal 10 μM SH-11037 or *t*-AUCB treatment effectively normalized CNV-induced sEH enzymatic activity and increased the ratio of 19,20-EDP to 19,20-DHDP, indicating that local pharmacological inhibition of sEH can alter the lipid metabolism in the eye (Sulaiman et al., 2018). These studies used 6–15 mice per treatment, plus vehicle injected controls (Basavarajappa et al., 2015; Sulaiman et al., 2016, 2018), which should be sufficient to avoid any confounding effects of inflammation, which is a concern with this delivery route (Chiu et al., 2007). An inhibitory effect on ocular angiogenesis was also reported for other routes of administration of sEH inhibitors. Oral administration of TPPU (1 mg/kg/day) not only reduced CNV lesions and vascular leakage, but also its coadministration with i.p. injection of 17,18-EEQ or 19,20-EDP (50 μg/kg/day) potentiated anti-angiogenic effects on CNV (Hasegawa et al., 2017). In addition, normal neonatal mice that received i.p. injection of *t*-AUCB (2 mg/kg, twice/day) from 1 to 4 days had significantly reduced retinal vascularization (Hu et al., 2014). Together, these studies support the therapeutic potential of sEH inhibitors for ocular neovascularization.

### Conflicts and Controversies

However, some studies do not support the finding that sEH inhibition reduces ocular angiogenesis (Gong et al., 2017). Shao et al. (2014) found that EDP is pro-angiogenic and inhibition of CYP epoxygenase rather than sEH reduced retinal neovascularization in OIR. Systemic overexpression of the CYP epoxygenase CYP2C8 and downregulation of sEH expression in the retina of OIR mice were reflected in the increased and decreased retinal EDP to DHDP ratio, respectively. Dietary ω-3 PUFAs enhanced OIR-induced retinal neovascularization in CYP2C8 overexpressing mice and reduced retinal neovascularization in sEH overexpressing mice. In the aortic ring assay, DHA had an anti-angiogenic effect which was abolished by CYP2C8 overexpression, whereas 19,20-EDP alone had no effect but 19,20-EDP + sEH overexpression reduced aortic sprouting (Shao et al., 2014). However, the macrovessels of the aortic ring do not fully recapitulate microvascular features of the choroid capillaries (Shao et al., 2013).

Panigrahy and colleagues showed that CYP2CJ and CYP2C8 overexpressing mice and *Ephx2*^-/-^ mice had enhanced corneal and neonatal retinal vascularization (Panigrahy et al., 2013) and enhanced tumor dependent corneal angiogenesis (Panigrahy et al., 2012). CYP2C8 inhibition with montelukast further inhibited OIR and L-CNV in mice fed with ω-3 and ω-6 PUFAs, as opposed to sEH inhibition with i.p. injection of UC1770 (0.3 mg/kg) which *enhanced* OIR and L-CNV (Gong et al., 2016a). Also, UC1770 promoted aortic ring and choroidal sprouting, while montelukast enhanced the anti-angiogenic effects of DHA on aortic and choroidal sprouting, and HREC proliferation, which were all rescued by 19,20-EDP (Gong et al., 2016a). CYP2C8 inhibition with fenofibrate also further reduced OIR and L-CNV in animals fed with dietary ω-3 PUFAs (Gong et al., 2016b). Again, 19,20-EDP reversed the anti-angiogenic effects of fenofibrate in aortic and choroidal sprouting, HREC migration, and tube formation, in contrast to DHA which enhanced fenofibrate’s anti-angiogenic effects (Gong et al., 2016b). Thus, these studies present conflicting results regarding sEH as a therapeutic target for ocular angiogenesis.

### Reconciling Disparate Findings

There are important factors to consider in attempting to integrate the conflicting experimental data: stability of EpFAs, routes of administration of CYP or sEH inhibitors and systemic or local modulation of sEH/CYP expression, modulation of other lipid mediator pathways, and experimental design considerations.

First, we cannot conclude that 19,20-EDP promotes ocular angiogenesis when studies added 19,20-EDP into growth media without modulating sEH activity in the aortic ring and choroidal sprouting assay (Gong et al., 2016a,b). Although 19,20-EDP is a fairly stable isomer, it can be converted into its corresponding diol 19,20-DHDP by sEH. Co-treatment with an sEH inhibitor is required to stabilize 19,20-EDP to ascertain that a change in phenotype is due to 19,20-EDP and to minimize effects from 19,20-DHDP that is generated by sEH activity. Indeed, Hasegawa et al. (2017) demonstrated greater inhibitory effects on L-CNV upon oral administration of sEH inhibitor TPPU + 17,18-EEQ or 19,20-EDP compared to 17,18-EEQ or 19,20-EDP alone. Additionally, aortic rings from *Ephx2*^-/-^ mice treated with 19,20-EDP or 17,18-EEQ did not show any effect on angiogenic sprouting. One explanation for this lack of effect is that the lipid concentration used (1 μM) (Shao et al., 2014) may be below the inhibitory threshold; further characterization of dose dependent effects of these compounds on angiogenesis may be necessary.

Second, the studies suggesting that inhibition of sEH promotes ocular angiogenesis employed sEH blockade via constitutive *Ephx2*^-/-^ mice (Panigrahy et al., 2013) or oral administration (Gong et al., 2016b) or systemic i.p. injection of sEH inhibitors (Gong et al., 2016a). It is difficult to compare such results to those from tissue specific local targeting of sEH, considering the potential confounding effect of the blood-retina barrier (BRB) and the unique retinal lipid profile. To validate sEH as a key player in ocular angiogenesis, local targeting of sEH (e.g., tissue-specific knockout, intraocular injection, or topical therapy) is necessary. Moreover, sEH inhibition can have opposite effects on angiogenesis depending on tissue levels of fatty acids that are parent to EpFAs. EETs and EDPs are the most abundant epoxy substrates of sEH in ω-6 ARA and ω-3 DHA rich tissues, respectively. EETs usually have proangiogenic effects (Pozzi et al., 2005; Michaelis et al., 2008; Xu et al., 2013) while EDPs have antiangiogenic effects (Zhang et al., 2013; Capozzi et al., 2014; Hasegawa et al., 2017; Hu et al., 2017). Systemic targeting of sEH could accumulate EETs and their effects on angiogenesis could outweigh those of EDPs. Conversely, local targeting of sEH in the eye would predominantly accumulate EDPs since ω-3 DHA is most enriched in retina (Querques et al., 2011). Likewise, levels of EDPs are substantially greater than EETs in retina (Hu et al., 2014).

Third, the studies that showed that DHA delivery and CYP inhibition are antiangiogenic do not necessarily lead to the conclusion that downstream EpFA metabolites are proangiogenic, since CYP inhibition and the resulting accumulation of DHA could lead to accumulation of metabolites from the COX and LOX pathway. The dietary intake of DHA reduced retinal (Connor et al., 2007; Stahl et al., 2010b; Shao et al., 2014; Gong et al., 2016b) and choroidal neovascularization (Moghaddam-Taaheri et al., 2011; Gong et al., 2016a,b), but the inhibition of CYP epoxygenase potentiated the beneficial effect of DHA against ocular angiogenesis, suggesting that the resulting accumulation of DHA or reduced generation of EDP or both are playing a role in ocular angiogenesis. In addition, the results can also be partially explained by studies that show DHA derived metabolites of LOX and COX such as resolvins and neuroprotectin are anti-inflammatory and inhibit retinal angiogenesis (Connor et al., 2007). It is likely that the beneficial effects of exogenous DHA plus CYP blockade are mediated by increasing free DHA that can efficiently compete with ARA for the two other major metabolic pathways, LOX and COX, which produce ARA-derived pro-inflammatory metabolites such as prostaglandins and leukotrienes (Calder, 2010). In addition, certain COX metabolites from EETs were shown to be pro-angiogenic (Rand et al., 2017). Even though inhibition of CYP would reduce the formation of EDP, the effect of increased DHA leading to reduction in COX/LOX-dependent ARA derived pro-inflammatory and pro-angiogenic metabolites, and increase in COX/LOX dependent DHA derived anti-inflammatory metabolites could outweigh the loss of EDP.

Finally, experimental design factors should be considered. The ω-3/ω-6 lipid composition of mouse chow can vary between facilities, potentially influencing findings. Sex differences in animal models matter, too, given the estrogen dependent suppression of sEH expression (Yang et al., 2018). This could possibly reduce the response to sEH loss/inhibition in female mice whereas response to sEH loss/inhibition could be more apparent in male mice. Among the studies discussed above, many did not specify the sex (Connor et al., 2007; Stahl et al., 2010b; Hu et al., 2014; Shao et al., 2014; Basavarajappa et al., 2015; Gong et al., 2016a,b) while some studies reported using male mice (Panigrahy et al., 2012, 2013; Yanai et al., 2014; Hasegawa et al., 2017) or female mice (Sulaiman et al., 2016, 2018), thus posing a challenge in assessing potential variability in results due to sex differences. In addition, sex differences and age are critical factors in the L-CNV model, as aged female mice (>9 months) develop more severe CNV lesions than age-matched male mice whereas sex differences are not significant in younger mice (Gong et al., 2015). Reassuringly, mice at 6–8 weeks of age were used in all relevant studies (Yanai et al., 2014; Gong et al., 2016a; Sulaiman et al., 2016, 2018), which is an ideal age range for the L-CNV model (Gong et al., 2015). Likewise, all studies showed rigor in use of littermate controls for the OIR model (Panigrahy et al., 2012; Shao et al., 2014; Basavarajappa et al., 2015; Gong et al., 2016a,b), which is important as mice from larger litters with poor postnatal weight gain develop more severe OIR (Stahl et al., 2010a; Kim et al., 2016).

## Non-Proliferative Diabetic Retinopathy

Soluble epoxide hydrolase has also been implicated in non-proliferative diabetic retinopathy. This early phase of the disease is characterized by pericyte loss and increased vascular permeability, distinct from the late, proliferative phase characterized by neovascularization (Hammes et al., 2002; Figure 1A). A recent study (Hu et al., 2017) reveals that increased retinal expression of sEH and corresponding production of 19,20-DHDP contribute to the progression of non-proliferative diabetic retinopathy in hyperglycemic *Ins2*^Akita^ mouse retinas and in the retinas and vitreous of human diabetic patients. Under normal conditions, retinal endothelial cells are connected by tight junction proteins and supported by pericytes. During the early phase of diabetic retinopathy, 19,20-DHDP alters the distribution of presenilin 1 in lipid rafts of the cell membrane, thereby preventing interaction between presenilin 1 and cadherins and disrupting endothelial cell to pericyte and endothelial cell-to-cell contacts. Treatment with sEH inhibitor *t*-AUCB (Table 1) in drinking water (2 mg/L) significantly reduced the retinal level of 19,20-DHDP and normalized vascular defects (reduced pericyte number, enhanced migration of vascular pericytes to the extravascular space, increased acellular capillaries and increased vascular permeability) that were present in the eyes of diabetic mice. Overexpression of sEH (delivered by intravitreal adenovirus) in retinal Müller glia increased retinal 19,20-DHDP and induced retinopathy in non-diabetic mice, highlighting that sEH may play a causative role in progression of the disease (Hu et al., 2017). This mechanism – disrupting endothelial cell junctions of the BRB by sEH-dependent production of 19,20-DHDP – is worth investigating further since defects in the BRB contribute to other eye diseases (Campochiaro et al., 1999; Green, 1999). Furthermore, a recent study reported that sEH inhibitor TPPU reduces fasting glucose level in rats (Minaz et al., 2018). Given this, the antihyperglycemic effect of sEH inhibitors in relation to diabetic retinopathy is also worth exploring.

## Diabetic Keratopathy

Diabetic keratopathy is characterized by delayed corneal epithelial wound healing and epithelial erosion, resulting in a compromised defense system against corneal injury and infective agents (Kaji, 2005). sEH is a potential therapeutic target for diabetic keratopathy, as tested in a mouse model where corneal erosions develop upon a single corneal debridement wound (Sun et al., 2018). The expression and enzymatic activity of sEH were increased in the corneal epithelial cells of streptozotocin-induced diabetic epithelial unwounded and wounded mice compared to control mice. *Ephx2*^-/-^ mice with streptozotocin-induced diabetes showed an increase in the rate of epithelial wound healing, decreased sensory nerve degeneration of corneas, and did not develop diabetes-associated dry eye symptoms. The loss of sEH also restored wound-induced STAT3 signaling and heme oxygenase-1 (HO-1) expression that were downregulated by hyperglycemic conditions. Similarly, sEH inhibition with subconjunctival 10 nM *t*-AUCB or clinical candidate inhibitor GSK2256294A (Table 1) promoted epithelial wound healing and restored HO-1 expression in diabetic mouse corneas (Sun et al., 2018).

## Uveitis

Uveitis refers to numerous intraocular inflammatory conditions often involving the uvea but not limited to this pigmented tissue layer (Brady et al., 2016; Figure 1A). Inhibition of sEH has anti-inflammatory effects in different models of inflammation (Askari et al., 2014; Hasegawa et al., 2017; Zhou et al., 2017). Given that a specific sEH inhibitor UC1728 (*t*-TUCB) (Table 1) had anti-inflammatory and analgesic effects in laminitic horses (Guedes et al., 2013), with a favorable pharmacokinetic profile in mice, it was proposed that subcutaneous injection of UC1728 might attenuate lipopolysaccharide (LPS)-induced inflammatory uveitis in rabbits (McLellan et al., 2016). In this model, *Escherichia coli*-derived LPS is injected into the anterior chamber (intracameral injection), inducing acute inflammation. Contrary to the hypothesis, treatment with UC1728 (3 mg/kg) was not efficacious in attenuating this uveitis. However, it is perhaps premature to conclude that sEH does not play a role in uveitis. Analysis of sEH expression and lipid profiles in the affected site may yet reveal sEH involvement that might respond to local treatment.

## Conclusion and Future Directions

Overall, there is strong evidence that stabilization of anti-inflammatory and anti-angiogenic EpFAs through sEH inhibition could be promising therapies for eye diseases. The unique anatomical and physiological features of the eye as a self-contained unit pose both advantages and disadvantages in drug discovery and delivery. Unlike other parts of the central nervous system, the eye is clinically accessible, allowing targeted drug delivery via routes of topical eye drops or intraocular injections, thus systemic side effects can be minimized. Conversely, barriers such as the cornea, blood aqueous-barrier and BRB hinder drug transport and absorption (Lee and Pelis, 2016). Therefore, implementing local routes of administration for lipid metabolites or sEH inhibitors in the eye is crucial, as is exploring the rich variety of sEH inhibitors that have been developed both preclinically and clinically (Shen and Hammock, 2012; Table 1).

Over the past decade, intravitreal anti-VEGF therapy has significantly advanced the treatment of neovascular eye diseases. But the only current delivery route for anti-VEGF agents, due to their large molecular weight, is intravitreal injection. This can be associated with intraocular inflammation, infection, hemorrhage, elevation of intraocular pressure, and cataract (Geroski and Edelhauser, 2000; Falavarjani and Nguyen, 2013), as well as patient inconvenience. Small molecule sEH inhibitors could provide advantages over anti-VEGF agents as they might be administered through non-invasive routes such as eye drops. Future studies may also reveal certain sEH inhibitors to be BRB permeable when delivered systemically, considering that a blood-brain barrier permeable sEH inhibitor, TPPU, has been characterized (Inceoglu et al., 2013).

Of course, adverse effects are still possible with sEH inhibitors if administered systemically, or if local delivery results in systemic exposure. Some of the compounds discussed here (such as TPPU and *t*-TUCB) have been tested for target specificity, with minimal non-specific binding to pharmacologically important proteins, rendering unexpected adverse effects unlikely (Lee et al., 2014) Nonetheless, it will also be important to assess the overall risk and benefit ratio of sEH inhibitors in the eye regardless of specificity. But polypharmacology can also offer therapeutic benefit: A COX-2/sEH dual inhibitor has been characterized as a potent agent against tumor angiogenesis and tumor growth (Wang et al., 2018). Utilization of such dual inhibitors or combined treatment of sEH inhibitors with other anti-inflammatory agents could also provide therapeutic potential against neovascular and inflammatory eye diseases. Understanding not only the biological activities of EpFAs and diols, but also the mechanisms by which they exert their biological effects in the eye is critical to develop sEH-mediated therapeutic approaches.

## Author Contributions

BP and TC wrote the manuscript, edited the manuscript, and approved the final version of the manuscript.

## Conflict of Interest Statement

TC is a named inventor on patent applications related to this topic and has received related research support from Inclera Therapeutics. The remaining author declares that the research was conducted in the absence of any commercial or financial relationships that could be construed as a potential conflict of interest.
